# Le syndrome Sturge-Weber: à propos d’un cas

**DOI:** 10.11604/pamj.2020.36.273.24346

**Published:** 2020-08-12

**Authors:** Meriem Doumiri, Mohamed Labied, Siham Salam, Dalal Laoudiyi, Kamilia Chbani, Lahcen Ouzidane

**Affiliations:** 1 Service de la Radiologie Pédiatrique, Hôpital Harouchi, Casablanca, Morocco

**Keywords:** Sturge-Weber, angiome plan, imagerie par résonnance magnétique, Sturge-Weber, planar angioma, MRI

## Abstract

Le syndrome de Sturge-Weber (SWS) ou angiomatose encéphalo-faciale, est un syndrome neuro-cutané et oculaire congénital rare. Il comporte deux types de malformations: capillaire faciale congénitale à type d’angiome plan et capillaro-veineux lepto-méningé de localisation le plus souvent pariéto-occipitale homolatérale. La Neuroimagerie, essentiellement l’imagerie par résonnance magnétique (IRM), joue un rôle important dans l'établissement du diagnostic, idéalement avant l'apparition de complications neuro-oculaires. Nous rapportons le cas d’un enfant chez qui le SWS est suspecté devant la présence d’un angiome facial et d’une épilepsie pharmaco-résistante.

## Abstract (en)

Sturge-Weber syndrome (SWS) or encephalofacial angiomatosis is a rare neurocutaneous and congenital ocular syndrome. It can cause two malformations: congenital facial capillary planar angioma and leptomeningal venous-capillary angioma (most often parieto-occipital homolateral angioma). Neuroimaging, in particular magnetic resonance imaging (MRI), plays an important role in the diagnosis, ideally before the occurrence of neuro-ocular complications. We report the case of a child in whom SWS was suspected based on facial angioma and pharmaco-resistant epilepsy.

## Introduction

Le syndrome de Sturge-Weber (SWS) ou angiomatose encéphalo-faciale, est un syndrome neuro-cutané et oculaire congénital rare. Il comporte deux types de malformations: capillaire faciale congénitale à type d’angiome plan et capillaro-veineux lepto-méningé de localisation le plus souvent pariéto-occipitale homolatérale. La tomodensitométrie et l’imagerie par résonnance magnétique sont considérées comme la clé diagnostic de ce syndrome.

## Patient et observation

**Figure 1:**
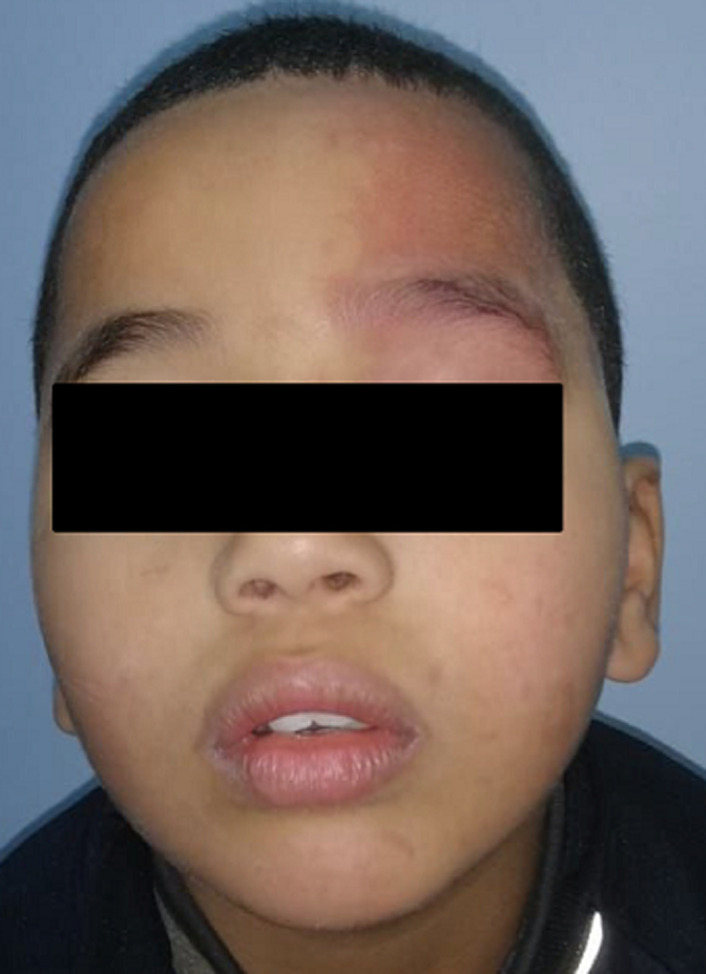
angiome plan fronto-orbitaire gauche

**Figure 2:**
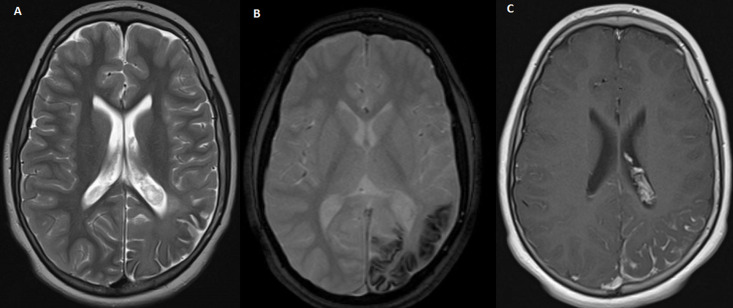
(A) séquence axiale T2: atrophie pariéto-occipitale gauche associée à une dédifférenciation substance blanche-substance grise; (B) séquence axiale T2(astérique): vides de signaux linéaires cortico-sous-corticaux en rapport avec des dépôts calciques; (C) séquence T1 écho de spin après injection de contraste: aspect épaissi du plexus choroïde gauche, et prise de contraste lépto-méningée en rapport avec un angiome pial

Nous rapportons le cas d’un enfant âgé de 6 ans, de sexe masculin, admis au service de radiologie pour le bilan d’une épilepsie pharmaco-résistante. On n'a pas noté d'épilepsie dans les antécédents familiaux, pas de notion de consanguinité, et l'enfant avait jusqu'à ce jour un bon développement psychomoteur. A l’examen, le patient était conscient, apyrétique, sans déficit sensitivo moteur. A l’examen clinique, on a objectivé un angiome plan cutané au niveau fronto-orbitaire gauche (Figure 1). Une IRM fut réalisée (Figure 2) mettant en évidence un épaississement du plexus choroïde gauche ainsi qu'une prise de contraste accentuée et gyriforme des sillons corticaux pariéto-occipitaux qui sont discrètement élargies témoignant de cette atrophie cérébrale homolatérale. Des dépôts calciques ont été détectées sur la séquence T2*. Ainsi le diagnostic d’un syndrome de Sturge Weber fut évoqué, et le patient a été adresser en consultation ophtalmologique.

## Discussion

Le syndrome de Sturge-Weber (SWS) est une phacomatose neuro-cutanée et oculaire, rare et sporadique, qui résulte d’une malformation du système vasculaire fœtal entraînant une anoxie corticale [1]. Dans sa forme complète ce syndrome comporte: une malformation capillaire faciale congénitale à type d’angiome plan, un angiome capillaro-veineux lepto-méningé de localisation le plus souvent pariéto-occipitale, avec atrophie cérébrale et calcifications sous-corticales, et des anomalies oculaires (glaucome et maladie vasculaire choroïdienne) [2,3]. L'anomalie physiopathologique fondamentale à la base de l'angiomatose leptoméningée est l'échec du développement normal des veines au niveau de la corticale et la persistance résultante du complexe vasculaire primitif fœtal [4]. Pendant de nombreuses années, ce syndrome était défini par une distribution selon le territoire du nerf trijumeau. Récemment, en 2014, Waelchli *et al*. ont proposé que la distribution de ce syndrome suit la distribution des vaisseaux sanguins embryonnaires du visage plutôt que celle du nerf trijumeau [5]. La présence de ces vaisseaux anormaux conduit à une altération de la perfusion cérébrale et à une ischémie progressive du parenchyme cérébral sous-jacent. Cela sera encore aggravé dans les cas de crises convulsives non contrôlées.

Dans 80% des cas, il existe une atteinte hémisphérique cérébrale unilatérale, ce qui explique la fréquence élevée d'hémiatrophie cérébrale chez les patients atteints du SWS [6]. Les manifestations cliniques du syndrome comprennent une épilepsie réfractaire au traitement, voire une hémiplégie, une hémianopsie et un retard mental. La neuroimagerie joue un rôle crucial dans l'établissement du diagnostic, idéalement avant l'apparition de complications neuro-ophtalmologiques. L’IRM aide à établir le diagnostic et à évaluer l’atteinte intracrânienne, mais il n’existe pas de consensus quant aux personnes à dépister, au moment optimal de l’imagerie, à la sensibilité de l’IRM ou à l’avantage global de l’identification des anomalies vasculaires à risque. La présence d'angiomatose leptoméningée a été considérée comme une caractéristique directe (impliquant généralement le cortex pariétal ou occipital postérieur ipsilatéral aux signes faciaux) [7]. L'IRM est la modalité d'imagerie idéale pour rechercher cette entité. Tout l'étendue de l'angiome pial est visualisé sous forme de prise de contraste dans les espaces sous-arachnoïdiens et des gyrus en regard.

L'atrophie des zones concernées caractérisent les cas avancés. La séquence pondérée en écho de gradient (T2*) est utile pour visualiser les dépôts calciques cortico-sous-corticaux. On peut retrouver un élargissement des veines profondes dû à une dysgénésie du système veineux superficiel, entraînant une dérivation du sang. Un épaississement du plexus choroïde peut également être retrouvé, attribué à cette dérivation dans les veines profondes [8]. Au cours des premières années de la vie, sur la séquence de perfusion, cette malformation vasculaire tend à devenir hypo perfusée, par altération du drainage veineux [9]. La tomodensitométrie montrent des calcifications dans la substance blanche sous-corticale et dans le cortex adjacent gyriforme (tram tracksign). La cause de ces calcifications est attribuée à une ischémie chronique due à un drainage veineux altéré. Un large spectre de malformations des développements corticaux est observé chez les patients atteints de SWS, allant de la polymicrogyrie et de la schizencéphalie à la dysplasie corticale focale [10].

## Conclusion

Le diagnostic du syndrome de Sturge-Weber est suspecté chez les patients présentant un angiome du front. Face à cette suspicion, un examen ophtalmologique et une IRM avec injection de gadolinium devraient être réalisés pour permettre un diagnostic précoce et réduire les complications ophtalmologiques et cérébrales.
